# Improving patient-centred decisions in severe aortic stenosis care

**DOI:** 10.1007/s12471-025-01940-9

**Published:** 2025-03-04

**Authors:** Judith J. A. M. van Beek-Peeters, Miriam C. Faes, Mirela Habibovic, Ben J. L. Van den Branden, Martijn W. A. van Geldorp, Nardo J. M. van der Meer, Mirella M. N. Minkman

**Affiliations:** 1 Department of Cardiothoracic Surgery, Amphia Hospital, Breda, The Netherlands; 2 Tilburg School of Social and Behavioural Sciences, Tilburg University, Tilburg, The Netherlands; 3 Department of Geriatrics, Amphia Hospital, Breda, The Netherlands; 4 Department of Medical and Clinical Psychology, Tilburg School of Social and Behavioural Sciences, Tilburg University, Tilburg, The Netherlands; 5 Department of Interventional Cardiology, Amphia Hospital, Breda, The Netherlands; 6 TIAS School for Business and Society, Tilburg University, Tilburg, The Netherlands; 7 Catharina Hospital, Eindhoven, The Netherlands; 8 Vilans, Centre of Expertise for Care and Support, Utrecht, The Netherlands

## Introduction

Decision-making in the treatment of older, symptomatic patients with severe aortic stenosis (AS) is complex due to multiple treatment options, patient characteristics and the personal preferences of both patients and professionals. Professional guidelines, including those from the Netherlands Society of Cardiology (*Nederlandse Vereniging voor Cardiologie*), and the Dutch Appropriate Care Framework emphasise the importance of shared decision-making (SDM) to integrate patient preferences and values, such as quality of life, into treatment decisions [1–5]. However, despite professionals’ willingness to adopt SDM, its implementation in daily practice remains a challenge.

## Benefits of and barriers to shared decision-making

SDM is a well-defined process in which patients and professionals discuss care and cure decisions based on the best available evidence and the patient’s personal preferences and goals [6]. Treatment options for severe AS in older patients include conservative treatment and transcatheter or surgical aortic valve replacement [3–5]. SDM empowers patients by involving them actively in their healthcare decisions, leading to more personalised and effective treatments. It is a key principle of sustainable, accessible and personalised healthcare in the Netherlands [2].

**Fig. 1:**
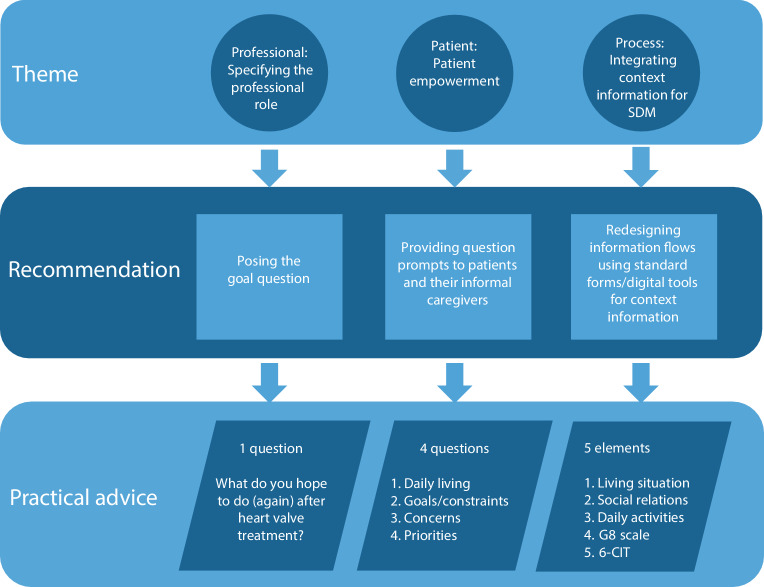
Key themes of shared decision-making (*SDM*) for patients with severe aortic stenosis, with recommendations and advice for professionals. (*6‑CIT* Six-Item Cognitive Impairment Test)

However, despite its many benefits, implementing SDM into daily practice remains difficult. Identification of SDM barriers has led to strategies to support professionals (e.g. training in SDM), patients and caregivers (e.g. decision aids) and systemic improvements (e.g. leadership, care pathways). While these strategies are helpful, they are not a quick fix. This article provides 3 key themes at the professional, patient and process levels and offers practical advice for professionals to incorporate SDM into their daily routines (Fig. 1).

## Key themes in shared decision-making

### Specifying the professional role

SDM is a collaborative process that helps professionals recognise the valuable knowledge patients and their caregivers contribute to high-quality decisions. It integrates the expertise of professionals with the unique perspectives of patients. While patients are not expected to be treatment experts, they are encouraged to share their values and preferences, to ensure the best AS treatment for their personal situation.

Research on a goal-oriented SDM model has shown that patients’ personalised treatment goals are often missing in decision-making for severe AS [7]. While real-world conversations may differ, starting with the patient’s treatment goals is essential. Professionals should simply ask their patients: ‘What do you hope to do (again) after heart valve treatment?’ (Fig. 1). This question helps to gather patient input, shifts the focus from the disease to personal goals and values and aids in setting realistic treatment expectations.

Before consultations, the responsibility of asking this goal question can be assigned to the professional, such as the cardiologist, nurse practitioner or nurse. It must be ensured that all professionals acknowledge the patient’s response, and clear communication protocols are needed for consistency and clarity. Since SDM is ongoing and treatment goals for severe AS may change, repeating the goal question throughout the patient journey is also required.

### Empowering patients

Effective SDM requires patients and their informal caregivers to take an active role in decision-making. Older patients, even those with low education levels or cognitive impairments, are often willing to participate in SDM when supported appropriately [8]. Informal caregivers can play a vital role in clarifying the patient’s personal contexts and facilitating communication with professionals.

Patient decision aids (PDAs) support SDM by guiding conversations between professionals and patients, but they must meet quality standards to be effectively implemented [8]. Integrating PDAs into workflows can be challenging, especially in cardiovascular practice. A Question Prompt List (QPL) is a structured tool to assess a patient’s personal situation and preferences and is designed to help patients prepare for medical consultations [8]. It includes a list of questions that patients can answer prior to these consultations. For severe AS, we are introducing a QPL with 4 key questions—covering daily living, goals/constraints, concerns and priorities—which can help patients define their treatment goals and engage more effectively in SDM (Fig. 1).

### Integrating context information for shared decision-making

Context information about a patient’s living situation, social relations, daily activities, frailty and cognitive status is often missing in care pathways [9]. This information is essential for tailoring treatment to individual needs. For example, in many heart centres, the treatment plan is developed without the physical presence of the referring cardiologist and without seeing the patient in the outpatient clinic beforehand, leading to decisions that may not align with the patient’s unique situation [9, 10].

To address this gap, professionals should redesign information flows to include context data as a standard part of the care process. This information can be obtained from other professionals involved in the patient journey, such as general practitioners and geriatricians, or may be collected by the referring cardiologist in collaboration with nurses and nurse practitioners. Context information to be shared should include 5 elements: the patient’s living situation, social relations, daily activities, G8 scale (for frailty) [11] and the Six-Item Cognitive Impairment Test (6-CIT) ([12]; Fig. 1). Standardised forms or digital tools can facilitate the collection and sharing of this information across the care pathway, ensuring that all professionals involved in the patient journey have access to relevant context data.

## Conclusion

As the population ages, effective SDM for severe AS requires professionals to prioritise understanding each patient’s goals, while patients must be supported in reflecting on their personal situations to define these goals. Redesigning information flows to include context data is crucial for ensuring that treatment decisions align with what matters most to patients. By addressing these key themes, SDM can bridge the gap between professional expertise and patients’ unique perspectives, thereby fostering patient-centred decisions.
